# Near‑Infrared Photobiomodulation in White‑Matter Disease: From Microglial States to Measurable Endpoints

**DOI:** 10.1007/s12017-026-08924-x

**Published:** 2026-06-30

**Authors:** Jiahao Zhang, Quanguang Zhang, J. Dedrick Jordan, Xuemei Zong

**Affiliations:** 1 Institute for Cerebrovascular and Neuroregeneration Research (ICNR), Department of Neurology, Louisiana State University Health Sciences Center, 1501 Kings Highway, Shreveport, LA 71103 USA; 2 Department of Neurology, College of Medicine - Tucson, The University of Arizona, Tucson, AZ 85724 United States

**Keywords:** Photobiomodulation, White matter, Microglia, Remyelination, Diffusion tensor imaging, Dosimetry

## Abstract

White-matter (WM) injury contributes to disability across multiple sclerosis, traumatic brain injury, Alzheimer’s disease and related dementias, and small-vessel disease. We use microglial state programs as an organizing axis for WM injury-to-repair logic, while emphasizing that WM outcomes are multicellular and involve oligodendrocyte-lineage cells, astrocytes, axons/neurons, and vascular factors. Microglia span an injury–repair continuum, from inflammatory programs that increase oxidative stress and debris burden to repair-competent programs that support debris handling, remyelination, and axonal integrity. Near-infrared photobiomodulation (PBM; ~800–1100 nm) is most consistently associated with modulation of mitochondrial redox/bioenergetic pathways and inflammatory tone. CCO-centered mechanistic framing is best established near ~ 800–850 nm, whereas longer wavelengths (e.g., ~ 1064–1070 nm) may involve additional initiating mechanisms with downstream convergence on shared redox/bioenergetic and inflammatory pathways. Across demyelination and spinal cord injury models, appropriately dosed PBM has been reported to reduce inflammatory glial readouts and to associate with improved myelin/axon-related endpoints and functional measures, although mechanistic certainty varies across models. Human evidence remains early but broadly supports safety; a randomized trial in moderate traumatic brain injury reported treatment-related changes in diffusion-MRI WM metrics, while small dementia and chronic-injury studies report heterogeneous cognitive and physiological signals. Given dose dependence and depth-limited transcranial delivery, we synthesize mechanism-informed, dose-aware reporting guidance and WM-anchored outcome frameworks that pair diffusion MRI/DTI with interpretable biomarkers (e.g., NfL, GFAP, sTREM2) and thermally controlled sham designs. We also note potential indirect/systemic contributions that could help reconcile depth–dose constraints with deeper WM effects.

## Introduction

WM pathology is a major contributor to disability across WM-dominant neurological diseases, including multiple sclerosis (MS) (Reich et al., 2018), traumatic brain injury (TBI) (Hulkower et al., 2013), and Alzheimer’s disease and related dementias (Garnier-Crussard et al., 2022), because demyelination and axonal injury disrupt saltatory conduction and large-scale network efficiency. In parallel, large-scale psychiatric imaging consortia have demonstrated reproducible WM microstructural differences across major psychoses, supporting WM integrity as a transdiagnostic substrate measurable by diffusion MRI (Kochunov et al., 2022; Thompson et al., 2020; Kelly et al., 2018). Diffusion tensor imaging (DTI) provides non-invasive microstructural indices: fractional anisotropy (FA) decreases with myelin/axonal disorganization, and mean diffusivity (MD) increases with tissue rarefaction. Large-scale consortia show widespread FA reductions in schizophrenia and other major disorders, while fornix and callosal FA reductions track cognitive decline in AD and mild cognitive impairment, supporting DTI as a quantitative WM endpoint for longitudinal studies and trials (Chen et al., 2023, Kantarci et al., 2014, Kelly et al., 2018).

Microglia are central to WM outcomes, but their responses span state continua rather than a strict M1/M2 dichotomy. Disease-associated microglia and white-matter-associated microglia (WAM) are transcriptionally defined programs engaged in neurodegeneration and aging WM, respectively, with signatures enriched for lipid handling, phagocytosis, and TREM2 signaling (Safaiyan et al., 2021, Deczkowska et al., 2018, Keren-Shaul et al., 2017). In demyelinating settings, microglia can amplify injury through NF-κB–associated cytokine programs (e.g., TNF and IL-1 family members). They can also enable repair by clearing inhibitory myelin debris and shaping a permissive milieu for oligodendrocyte progenitor cell (OPC) maturation and remyelination (Cignarella et al., 2020) (Miron et al., 2013). Recent mouse and human data further link resident microglia to the maintenance of adult myelin integrity, supporting a causal role for microglial dysfunction in progressive WM vulnerability (Mcnamara et al., 2023). Axonal degeneration—often via Wallerian-like, mitochondria-linked programs—can then compound demyelination and drive disconnection syndromes that underlie progressive deficits (Wang et al., 2021a, Coleman & Höke, 2020).

Photobiomodulation (PBM) uses red/near-infrared light to modulate cell physiology at non-thermal doses. Mechanistic evidence is strongest for mitochondrial and redox-linked pathways. CCO-centered initiation models are best established in the ~ 800–850 nm range (e.g., ~ 810 nm), whereas longer NIR wavelengths (e.g., ~ 1064–1070 nm) may involve additional initiating mechanisms; downstream signaling can converge on shared redox/NO-related and bioenergetic nodes (de Freitas & Hamblin, 2016, Hamblin, 2018). At the same time, specific upstream steps—particularly direct, in vivo photochemical effects on CCO—remain debated, underscoring the need to distinguish established mechanisms from plausible but still inferred pathways (Quirk & Whelan, 2020). PBM responses are biphasic (Arndt–Schulz): insufficient fluence is ineffective, whereas excessive exposure can blunt or reverse benefits. Therefore, explicit reporting of wavelength, irradiance, spot size/coverage, session duration, and per-session fluence (J/cm²) is essential for reproducibility and biological interpretation (Zein et al., 2018, Huang et al., 2011). For transcranial PBM, depth–dose attenuation through scalp and skull further constrains target engagement, and wavelength plus beam geometry strongly influence delivered energy to cortical and subcortical regions (Tedford et al., 2015).

Because PBM efficacy is dose-dependent and transcranial target engagement is depth-limited, WM translation requires WM-anchored endpoints and WM-relevant microglial mechanisms. Microglia contribute to WM repair through myelin debris clearance/lipid handling, support of OPC maturation and remyelination, and preservation of axonal health. Preclinical studies suggest PBM can reduce neuroinflammatory signaling and bias glial programs toward repair, but direct evidence for each WM-specific microglial function remains uneven. Proposed pathways such as meningeal lymphatic/glymphatic facilitation are supported mainly by early or indirect evidence (Semyachkina-Glushkovskaya et al., 2020). Human evidence remains limited, with one moderate TBI trial reporting feasibility/safety and DTI changes, while dementia studies are small and heterogeneous (Figueiro Longo et al., 2020). Accordingly, WM-endpoint–anchored trials with harmonized dosimetry and paired WM imaging plus microglial biomarkers are needed.

## Microglial State Programs in White-Matter Injury and Repair

**Fig. 1:**
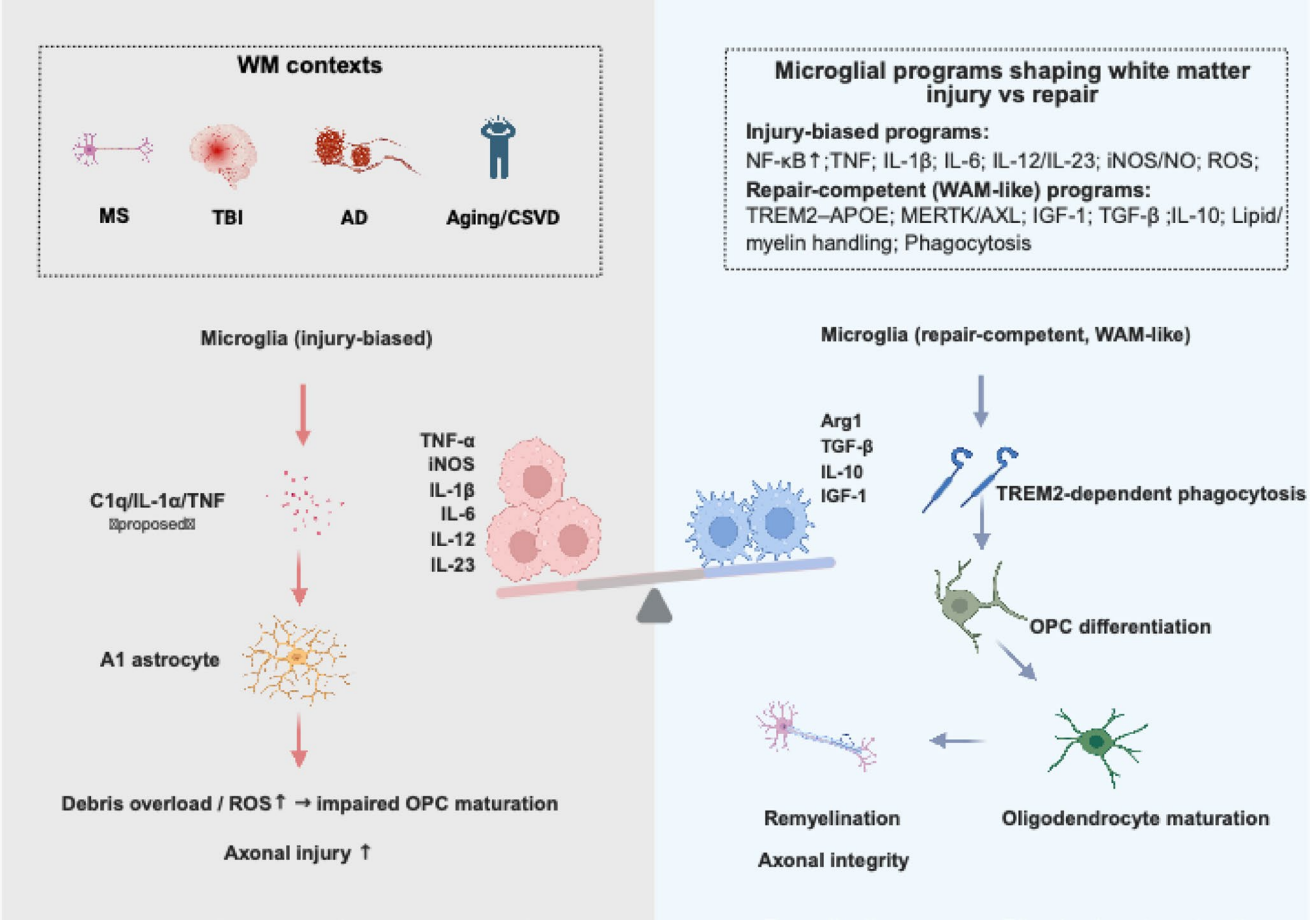
WM contexts and microglial programs along an injury–repair continuum. Left: Across MS, TBI, AD, and aging/small-vessel disease, injury-biased glial/myeloid programs are associated with inflammatory and oxidative stress signaling, increased debris burden, impaired OPC maturation, and axonal vulnerability. Microglia–astrocyte cues (e.g., C1q/IL-1α/TNF) have been proposed to promote neurotoxic astrocyte programs in some contexts. Right: Repair-competent WAM-like programs support debris clearance/lipid handling and pro-resolving signaling, promoting OPC differentiation, remyelination, and axonal integrity. The balance depicts a continuum rather than a strict M1/M2 dichotomy. This schematic is a representative organizing framework and does not imply microglia-exclusive causality

Microglia occupy a continuum of functional states shaped by local cues of injury and altered homeostasis rather than a simple M1/M2 binary. In this review, microglial state programs are used as an organizing axis for WM injury-to-repair logic; however, WM outcomes arise from coordinated contributions of multiple cell types (including oligodendrocyte-lineage cells, astrocytes, and axons), and microglial signatures are not necessarily WM-exclusive without spatial and cell-type–resolved validation. Single-cell studies have identified transcriptional programs such as disease-associated microglia and WAM(-like) states, often enriched for phagocytosis and lipid/myelin processing in neurodegeneration and aging WM (Safaiyan et al., 2021, Keren-Shaul et al., 2017). In WM injury, microglia can act as both effectors of damage and facilitators of repair. Across demyelinating models, pro-inflammatory programs can exacerbate tissue injury, whereas repair-competent programs promote clearance of inhibitory myelin debris and support oligodendrocyte lineage progression (Miron et al., 2013). Figure 1 summarizes this injury-to-repair axis and highlights three WM-relevant microglial functions that organize downstream outcomes: myelin debris clearance/lipid handling, support of OPC maturation and remyelination, and preservation of axonal health.

As shown in Fig. 1, injury-biased programs characterized by NF-κB–linked cytokine signaling and iNOS/ROS pathways can increase oxidative stress and debris burden, thereby constraining OPC maturation. In contrast, repair-competent (WAM-like) programs engage TREM2-dependent phagocytosis and lipid/myelin handling and provide pro-resolving mediators that support OPC differentiation, oligodendrocyte maturation, remyelination, and axonal integrity. Microglia–astrocyte signaling may further shape WM outcomes. Complement/cytokine cues (e.g., C1q/IL-1α/TNF) have been proposed to promote neurotoxic astrocyte programs in inflammatory contexts, although the in vivo contribution can vary across models and disease stages (Liddelow et al., 2017).

Across disease settings, microglial and broader myeloid/glial activation relevant to WM injury is frequently observed but differs in timing, anatomical distribution, and state programs. In multiple sclerosis, microglial activation is detected in lesions and in normal-appearing WM, and chronic active lesions are associated with ongoing inflammation and worse outcomes (Polvinen et al., 2023, Bagnato et al., 2024). In injury settings including TBI and focal cortical injury, single-cell and spatial profiling studies show persistent injury-responsive glial/myeloid programs accompanying diffuse axonal injury and delayed myelin degeneration; however, these datasets are not WM-restricted and should not be cited as direct evidence for WM-selective microglia (Koupourtidou et al., 2024, Jha et al., 2024). In aging and cerebral small vessel disease (CSVD), WAM-like signatures have been reported in vulnerable WM regions, and emerging human studies suggest associations between impaired perivascular/lymphatic clearance and WMH burden; however, causal relationships remain to be established (Safaiyan et al., 2021, Zhou et al., 2024). In AD, TSPO-PET provides an in vivo readout of glial/innate immune activation (not microglia-specific) that has been associated with pathology and cognitive impairment, while the spatial and temporal relationship to WM injury remains under active investigation (Rossano et al., 2024). Accordingly, TSPO-PET should be interpreted as a glial/innate immune marker with potential contributions from activated microglia, infiltrating macrophages, and reactive astrocytes; we therefore avoid microglia-exclusive attribution from TSPO alone and emphasize multimodal triangulation (e.g., TSPO-PET with diffusion MRI/DTI and fluid biomarkers) to improve interpretability.

Biomarkers and imaging provide complementary tools to map these processes. IBA1 labels microglial lineage, CD68 reflects lysosomal/phagocytic activity, and TMEM119 and P2RY12 help distinguish resident microglia from infiltrating macrophages (Lier et al., 2021, Kenkhuis et al., 2022). TSPO-PET enables in vivo mapping of neuroinflammation but is not microglia-specific and requires careful interpretation. First-generation tracers have limited signal-to-noise, whereas second-generation tracers are influenced by rs6971 genotype and binding affinity, motivating genotype correction in analyses (Mizrahi et al., 2012, Salerno et al., 2024). DTI complements molecular imaging by quantifying WM microstructural injury, and multimodal studies have linked inflammatory biomarker panels and TSPO-PET signals to reduced FA and related metrics, supporting an association between glial activation and WM integrity (To et al., 2023, Bagnato et al., 2024).

Together, current evidence supports a working model in which sustained injury-biased myeloid/glial activation—often involving microglia in concert with other WM-resident and infiltrating cell types—maintains cytokine/oxidative stress programs, limits effective debris clearance, and constrains OPC maturation, thereby amplifying demyelination and axonal vulnerability. Strategies that bias the balance of glial/myeloid programs toward repair-competent states—and test these shifts using WM-anchored imaging endpoints alongside appropriately interpreted glial biomarkers—represent a plausible translational direction for WM-dominant disease settings.

## Mechanisms of Near-Infrared Photobiomodulation in Microglial Modulation

**Fig. 2:**
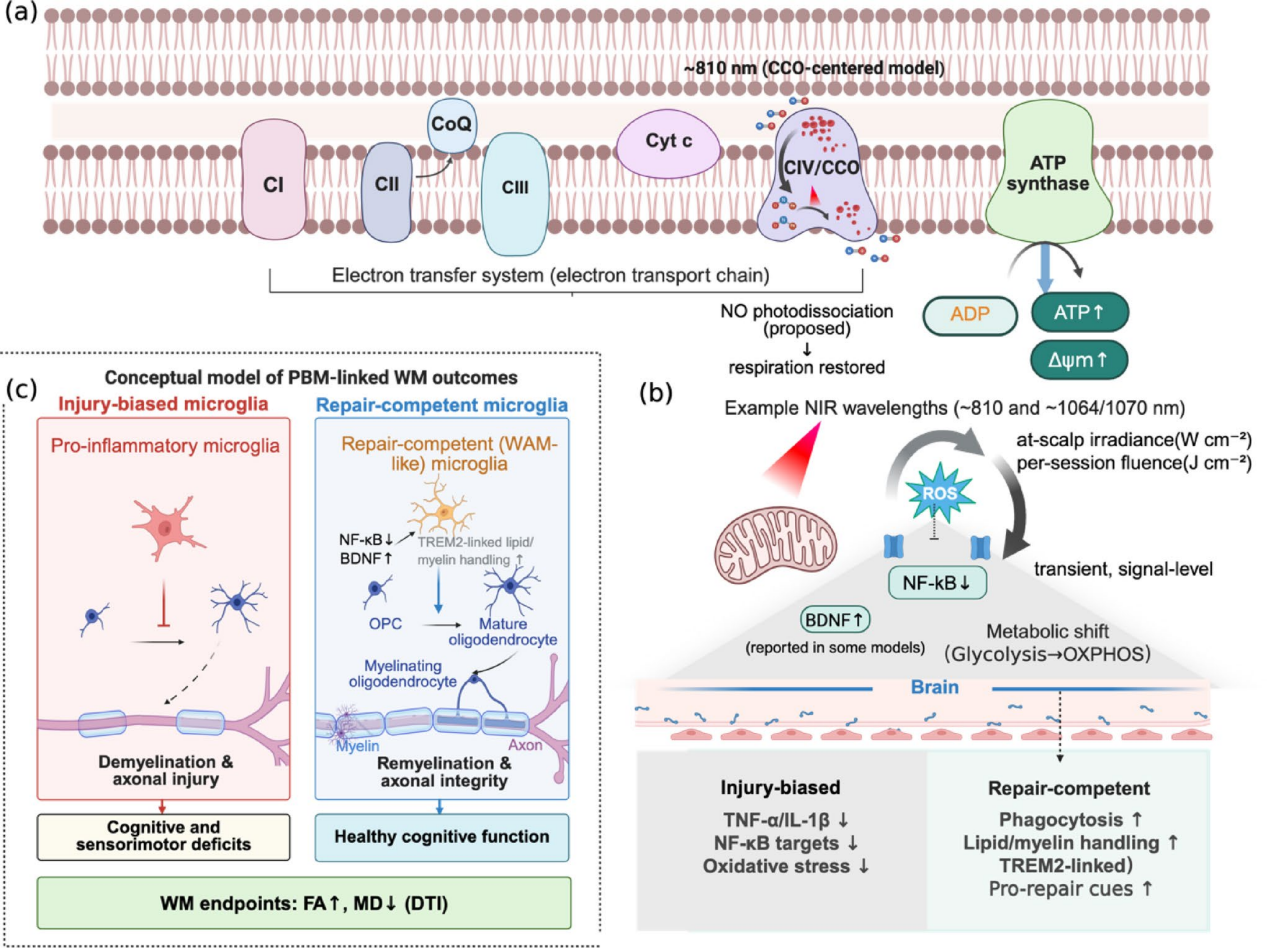
From mitochondrial photobiology to microglial state shifts and WM endpoints under near-infrared PBM. (**a**) Initiation and bioenergetic signaling: CCO-centered models are best established near ~ 800–850 nm (e.g., ~ 810 nm), where PBM has been associated with changes in Δψm/ATP and transient ROS; NO photodissociation from CCO has been proposed in some models. Longer wavelengths (e.g., ~ 1064–1070 nm) may involve additional initiating mechanisms with downstream convergence on redox and inflammatory pathways. (**b**) Dose-aware signaling: At-scalp irradiance and per-session fluence map to downstream readouts (e.g., NF-κB–linked signaling and BDNF in some models) and metabolic shifts toward oxidative phosphorylation. (**c**) Myeloid/glial and WM outcomes: PBM has been reported to bias glial/myeloid programs toward repair-competent states (WAM-like), increasing phagocytosis and lipid/myelin handling (TREM2-linked) and supporting OPC differentiation, remyelination, and axonal integrity. WM endpoints referenced include diffusion-MRI/DTI metrics (e.g., FA, MD). Elements shown are representative rather than exhaustive.

Near-infrared PBM (~ 800–1100 nm) is most consistently linked to modulation of mitochondrial redox and bioenergetic tone. Cytochrome-c oxidase (CCO) is a leading candidate photoacceptor, and CCO-centered mechanistic framing is best established in the ~ 800–850 nm range (e.g., ~ 810 nm); however, the contribution of direct CCO photochemistry to in vivo effects is not fully resolved (de Freitas & Hamblin, 2016, Hamblin, 2018). Across cell and tissue models, PBM has been reported to increase mitochondrial membrane potential (Δψm) and ATP availability and to elicit transient, signal-level ROS that can engage downstream transcriptional programs. PBM-mediated relief of nitric-oxide–linked respiratory inhibition via CCO photodissociation has also been proposed, with the strength of evidence varying by model and experimental context (de Freitas & Hamblin, 2016, Hamblin, 2018). Importantly, initiating mechanisms may be wavelength-dependent: for longer NIR wavelengths (e.g., ~ 1064–1070 nm), additional initiation hypotheses have been proposed, with potential downstream convergence on shared redox/bioenergetic and inflammatory pathways. These wavelength-dependent initiation models and convergent downstream signaling pathways are summarized in Fig. 2a–b.

Downstream of these primary events, PBM can bias inflammatory signaling in a context-dependent manner. In several neuroinflammatory settings, PBM has been associated with reduced NF-κB activity and altered cytokine output, and in some brain models PBM has been reported to increase neurotrophin signaling (e.g., BDNF) and pro-survival pathways (e.g., PI3K–Akt), which may indirectly shape glial function and tissue resilience (Ma et al., 2022, Xuan et al., 2015). Because these readouts are sensitive to dose and delivery, Fig. 2b conceptually links at-scalp irradiance and per-session fluence to representative downstream markers (e.g., NF-κB and BDNF) and to a metabolic shift toward oxidative phosphorylation.

A key translational question for WM is whether PBM modulates microglial functions that directly govern repair: myelin debris clearance/lipid handling, support of OPC maturation and remyelination, and preservation of axonal integrity (Fig. 2c). Preclinical studies provide convergent but heterogeneous evidence that PBM can temper chronic, injury-biased microglial reactivity while preserving or enhancing phagocytosis and metabolic homeostasis. For example, in Aβ-challenged microglia, 808 nm PBM reduced TNF-α and IL-1β and increased phagocytosis, alongside a shift from glycolysis toward mitochondrial respiration, improving neuronal survival in co-culture (Stepanov et al., 2022). In injury models, PBM has been reported to attenuate pro-inflammatory innate immune–glial signaling and inflammasome-associated pathways, accompanied by functional improvement (Wang et al., 2021b ,Ma et al., 2022). Because the responding myeloid compartment can include both resident microglia and infiltrating macrophages depending on model context, cell-type–resolved readouts are required for precise attribution. In additional injury-adjacent settings (e.g., postoperative neurocognitive disorder), NIR PBM has also been associated with neuroprotective outcomes alongside changes in immune signaling (Zhang et al., 2024a). Importantly, not all studies directly measure WM-relevant myeloid/glial functions (e.g., myelin debris clearance or lipid handling), and mechanistic links to remyelination are sometimes inferred from marker changes rather than functional assays.

Beyond microglia, PBM can also act on the broader glial–immune milieu and on oligodendrocyte lineage biology, which is highly relevant to WM repair. In the cuprizone demyelination model, transcranial 808 nm PBM mitigated myelin loss and increased oligodendrocyte-lineage markers, consistent with enhanced survival and maturation (Duarte et al., 2018). In experimental autoimmune encephalomyelitis (EAE), 670 nm or dual 660 + 850 nm PBM reduced glial reactivity and axonal degeneration within spinal WM (Escarrat et al., 2024, Muili et al., 2012). After spinal-cord injury, PBM restrained astrocytic reactivity and extracellular matrix inhibitors and limited pSTAT3 signaling, supporting axon growth and preservation of WM architecture (Sun et al., 2020, Wang et al., 2021b, Zhang et al., 2024b). These studies collectively support anti-inflammatory and metabolic reprogramming as recurring themes, while highlighting that cell-type specificity (microglia vs. oligodendrocyte lineage vs. astrocytes) can differ by model and dose.

PBM responses are dose-dependent and are often described as biphasic (Arndt–Schulz-type): in many systems, low-to-moderate energy densities are associated with beneficial effects, whereas higher doses can attenuate or even reverse these responses (Huang et al., 2011, Huang et al., 2009). For transcranial delivery, tissue optics constrain target engagement; only a small fraction of incident NIR reaches cortex, and estimates vary by wavelength, site, and method (Henderson & Morries, 2015, Tedford et al., 2015, Jagdeo et al., 2012). Accordingly, mechanistic interpretation and cross-study comparison require consistent reporting of wavelength, irradiance, fluence, pulse structure, spot size/coverage, session duration, treatment schedule, and—when possible—estimated intracranial dose. Harmonized dosimetry is particularly important when WM endpoints (e.g., DTI metrics) are used to bridge preclinical mechanisms to clinical translation.

## Preclinical and Clinical Evidence for PBM in White Matter Protection

Across preclinical models, red/near-infrared PBM has been reported to reduce neuroinflammatory signaling and to improve WM-relevant histological or functional readouts. However, the extent to which these effects are mediated specifically by microglia—particularly via WM-critical functions such as myelin debris clearance/lipid handling, support of OPC maturation/remyelination, and preservation of axonal integrity—varies by model, delivery geometry, and the assays used.

In spinal-cord injury models with direct WM tract damage, epidural/implant-based 810 nm regimens were reported to suppress Lcn2–JAK2/STAT3-linked glial stress programs, reduce neurotoxic glial signatures, decrease apoptosis, preserve tissue, and improve locomotion (Wang et al., 2021b). In toxin-induced demyelination, a transcranial 808 nm regimen (≈ 36 J cm⁻² per session; six sessions) attenuated cuprizone injury, increased oligodendrocyte-lineage markers, and reduced microglial/astrocytic activation alongside motor improvement (Duarte et al., 2018). Related spinal-cord studies report reductions in astrocytic reactivity and CSPG deposition (often associated with lower pSTAT3 signaling) and shifts in macrophage/microglia phenotype markers, with improved motor recovery (Song et al., 2017; Sun et al., 2020). Mechanistic links have been proposed for nodes such as Notch1–HIF-1α/NF-κB, STAT3-linked pathways, and TLR2/NLRP3-related signaling. However, these pathways are frequently model- and dose-dependent and are not uniformly tied to WM-repair-specific functional assays (Zuo et al., 2025, Ju et al., 2023a, Ju et al. 2023b, Zhang et al., 2023, Ma et al., 2022). In autoimmune demyelination, 670 nm PBM reduced disease severity and was associated with remyelination-supportive outcomes (Muili et al., 2012). Table 1 summarizes PBM regimens and WM-relevant outcomes across these preclinical models.

Beyond classical WM-injury paradigms, AD and vascular studies provide mechanistic signals that may be relevant to WM vulnerability, but WM-specific endpoints are not consistently measured. For example, pulsed 1070 nm PBM increased microglial contact with amyloid, reduced plaque burden, and improved memory in AD models, with additional vascular readouts in some studies (Tao et al., 2021). Stroke preconditioning studies report improved cerebral blood flow with eNOS-linked signaling (Yokomizo et al., 2024). These findings motivate WM-focused testing, but should be interpreted as indirect with respect to WM repair unless WM-specific imaging or histology is included.

Early clinical data support safety and provide WM-relevant signals, with the strongest WM-anchored evidence coming from diffusion-MRI. In a randomized, double-blind, sham-controlled trial of moderate TBI, treatment within 72 h using a helmet-based NIR array (three × 20-min sessions) produced significant time-by-treatment interactions across diffusion-MRI WM metrics at three months, while symptom scores did not differ (Figueiro Longo et al., 2020). In dementia and MCI, small studies report cognitive, perfusion, fNIRS, or rs-fMRI network changes after transcranial (± intranasal) PBM, but WM-specific imaging is heterogeneous or absent across trials (Chan et al., 2024, Chan et al., 2021, Carneiro et al., 2019, Nizamutdinov et al., 2021, Chao, 2019). Table 2 summarizes clinical PBM studies and explicitly distinguishes WM-specific imaging from non-WM endpoints.

**Fig. 3:**
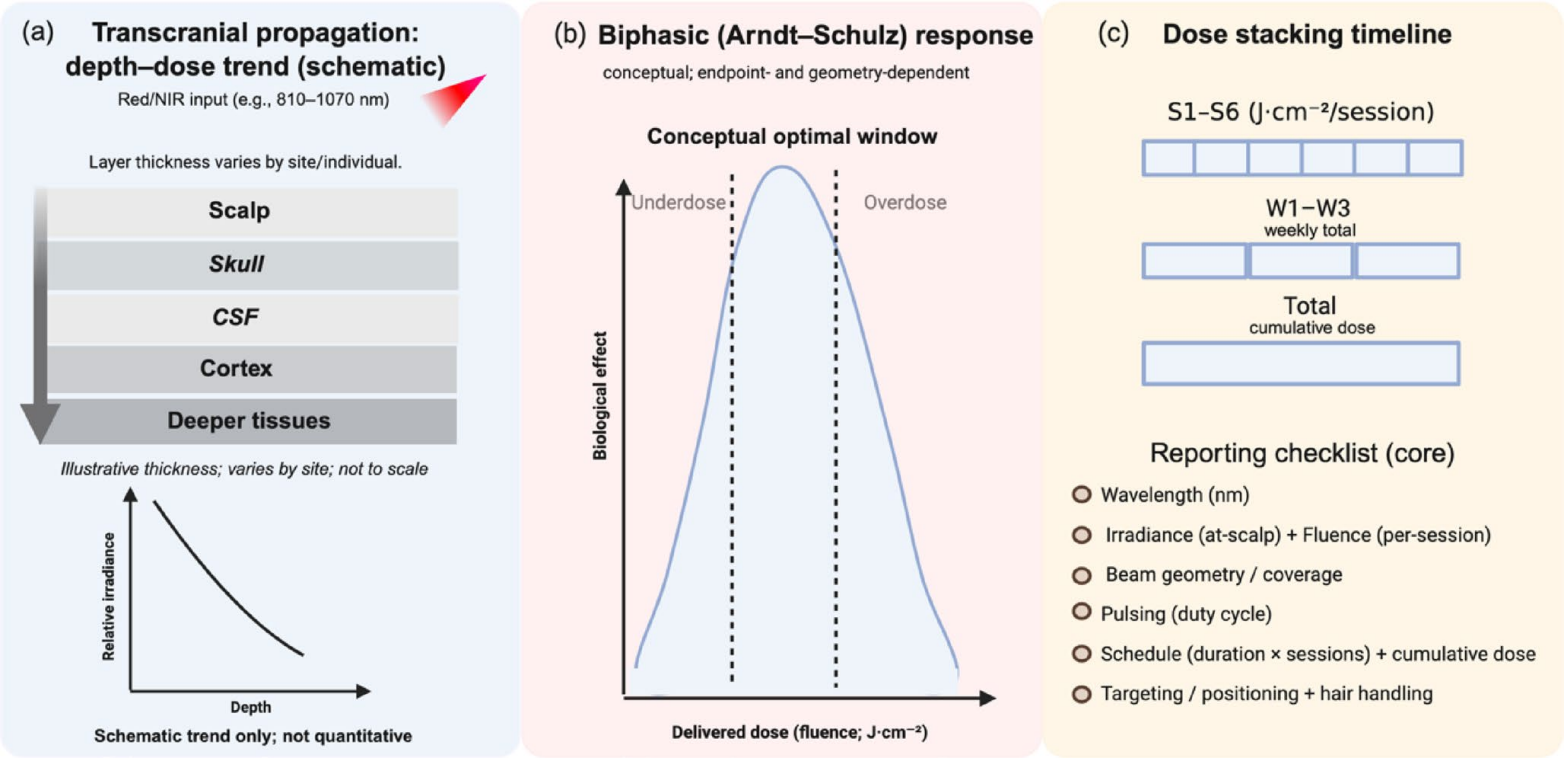
Transcranial delivery and dosing principles relevant to WM-anchored PBM studies. (**a**) Depth–dose trend (schematic): Red/NIR input (e.g., 810–1070 nm) is attenuated with increasing depth across scalp, skull, CSF, cortex, and deeper tissues. Layer thickness varies by site and individual; the panel illustrates a qualitative trend rather than a quantitative anatomical or optical model. (**b**) Biphasic response: Conceptual Arndt–Schulz-type relationship in which biological effects peak within an optimal dose window; the window is endpoint-, tissue-, wavelength-, and delivery-geometry–dependent. (**c**) Dose stacking and core reporting checklist: Cumulative exposure accrues across sessions and weeks. Core reporting items include wavelength; at-scalp irradiance and per-session fluence; beam geometry/coverage; pulsing (duty cycle); schedule (duration × sessions) with cumulative dose; and targeting/positioning with hair handling

Taken together, current animal and human evidence suggests that PBM can modulate inflammatory and metabolic states in ways that may favor WM resilience. At present, direct demonstrations that PBM enhances WM-critical microglial functions (e.g., myelin debris clearance/lipid handling) in vivo—and that these changes causally drive remyelination—remain limited. Therefore, a rigorous translational path is WM-endpoint-anchored trial design with harmonized dosimetry (wavelength, irradiance, fluence, beam geometry/coverage, pulsing, schedule, and estimated intracranial dose when feasible). Such studies should incorporate paired biomarker readouts that can test glial engagement (e.g., TSPO-PET with tracer/genotype considerations, alongside fluid markers). Figure 3 summarizes depth-limited transcranial delivery, biphasic dose response, and a reporting checklist; Table 3 maps WM pathophysiological nodes to PBM-relevant mechanisms with evidence tags.

## Challenges, Limitations, and Future Directions

Despite growing preclinical and early clinical interest, clinical translation of photobiomodulation for white-matter protection remains constrained by gaps in target engagement, mechanistic specificity, and clinical endpoint alignment. Current evidence is most consistent with PBM acting as a modulator of mitochondrial/redox biology and inflammatory tone across multiple models, but direct demonstration that PBM reliably improves WM outcomes through WM-critical microglial functions—such as myelin debris clearance/lipid handling, support of oligodendrocyte lineage progression, and preservation of axonal integrity—remains uneven across studies.

A major limitation is the limited duration and heterogeneity of human evidence. Many clinical studies are short and use variable cognitive or network-level readouts, making it difficult to infer sustained effects on chronic WM pathology in disorders such as AD or multiple sclerosis (Huang et al., 2024). In parallel, PBM parameters vary widely across trials and preclinical work (wavelength, irradiance, fluence, pulsing, beam geometry/coverage, and treatment schedule). Because PBM responses can be biphasic, incomplete or inconsistent reporting of these parameters can plausibly contribute to variable efficacy and complicate quantitative synthesis (Shen et al., 2024, Nizamutdinov et al., 2021). Standardized dosimetry reporting and explicit definition of the delivered dose at the tissue of interest are therefore essential.

Target engagement is a specific challenge for WM translation. Transcranial light is strongly attenuated by scalp and skull, and cadaver studies and computational modeling suggest that only a small fraction of incident energy reaches intracranial tissue, with substantially lower delivery expected at increasing depth (Henderson & Morries, 2015, Tedford et al., 2015). This optical constraint does not rule out clinically meaningful effects, but it requires careful interpretation of “deep WM engagement” claims and motivates (i) explicit reporting of at-source and at-scalp irradiance, spot size/coverage and beam geometry, pulsing, and session duration; and (ii) optical modeling or empirical measurements to estimate intracranial dose where feasible. It also motivates consideration of indirect mechanisms that do not require direct deep-WM photon delivery.

While depth-limited transcranial delivery constrains direct photon–target engagement in deep WM, subcortical effects reported in some studies could also reflect indirect or systemic (“abscopal”) pathways. Proposed routes include modulation of circulating immune cells and blood cells, as well as blood-borne mediators (e.g., cytokines or extracellular vesicles), vascular/NO-related biology, or systemic metabolic/redox tone that secondarily shapes neuroinflammation and WM vulnerability. These mechanisms are not mutually exclusive with local cortical engagement and provide testable alternatives to reconcile the depth–dose constraint with downstream WM signals. In future trials, pairing WM imaging with peripheral readouts (e.g., immune phenotyping, inflammatory proteomics, and redox/NO-related markers) could help distinguish local versus systemic contributions.

Preclinical evidence also has important constraints. Typical group sizes are modest, which limits power in heterogeneous CNS injury and neurodegeneration models. Outcome measures vary widely across studies (behavioral tests, histology, cytokine panels), reducing cross-study comparability. Critically, WM-anchored endpoints are not consistently integrated, and microglial mechanisms are often inferred from marker changes rather than functional assays of debris clearance, lipid handling, or support of oligodendrocyte lineage progression (Qian et al., 2025). In addition, rodent cranial optics differ substantially from humans, and higher relative penetration can overestimate transcranial target engagement; species differences in WM composition and microglial dynamics further limit direct extrapolation (Liebert et al., 2023). These limitations, noted in recent evidence syntheses, underscore the need for methodological refinement and more explicit WM-focused endpoints (Ding et al., 2024).

Future work should prioritize WM-anchored, mechanism-testable designs. First, larger randomized controlled trials should pre-specify WM-relevant imaging endpoints (DTI metrics such as FA/MD and, where available, myelin-sensitive measures) and harmonize PBM dosimetry and delivery geometry across sites (Nairuz et al., 2024). Sham devices should be thermally neutral (matched contact temperature/heat flux and sensory cues without photon delivery), with scalp temperature monitoring and prespecified thermal equivalence criteria to control heating-related confounds, particularly when diffusion-MRI/DTI endpoints are used. Second, studies should pair WM imaging with interpretable biomarkers, recognizing specificity limits (e.g., TSPO-PET reflects neuroinflammation but is not microglia-exclusive). Actionable fluid candidates include NfL (axonal integrity), GFAP (astroglial reactivity), and sTREM2 (myeloid/TREM2-linked activity relevant to myelin-debris handling), sampled longitudinally alongside imaging. Third, preclinical work should directly test WM-critical myeloid/glial functions (debris phagocytosis, lipid handling, links to OPC maturation/remyelination and axonal integrity) with dose–response experiments that quantify target engagement. Where subcortical WM signals are observed despite depth–dose constraints, adding peripheral immune/redox readouts can help evaluate potential indirect/systemic contributions. Finally, combination and personalization strategies (including home-use or wearable devices) should be evaluated with WM-anchored endpoints, harmonized dosimetry, and safety monitoring rather than assumed from feasibility alone (Saied et al., 2019, Zhang et al., 2025, Liu et al., 2023). Taken together, these steps aim to convert promising mechanistic signals into reproducible, clinically meaningful WM outcomes.

## Conclusion

Near-infrared photobiomodulation is a promising, biologically motivated approach for supporting white-matter integrity in disorders where glial and myeloid programs—including microglia—contribute to injury propagation and repair in coordination with oligodendrocyte-lineage cells, astrocytes, and axons/neurons. Across experimental systems, the most consistent mechanistic signals point to modulation of mitochondrial/redox biology and nitric-oxide–linked pathways, with downstream effects on cellular energy status and inflammatory tone. Preclinical studies further suggest that PBM can bias glial programs toward more repair-permissive states; however, direct in vivo evidence that PBM reproducibly enhances WM-critical microglial functions—such as myelin debris clearance/lipid handling, support of oligodendrocyte lineage progression, and preservation of axonal integrity—remains variable and is not uniformly demonstrated across models and assays.

Evidence to date supports feasibility and biological activity, but clinical conclusions remain preliminary. In animal models, appropriately dosed regimens have been associated with reduced demyelination and improved functional readouts, while early human studies—including a randomized trial in traumatic brain injury—suggest safety and provide WM-relevant signals on diffusion MRI, with clinical endpoints still heterogeneous across cohorts. The next priority is rigorous, WM-anchored clinical testing with harmonized dosimetry and explicit target engagement considerations. Prospective trials should pre-specify wavelength, delivery geometry/coverage, at-scalp irradiance, per-session fluence, pulsing parameters, and treatment schedule (including cumulative exposure), and incorporate WM-specific imaging endpoints (e.g., diffusion metrics and, where feasible, complementary myelin-sensitive measures). Pairing WM imaging with biomarkers of glial activity—interpreted with appropriate specificity constraints—will be essential to test mechanism in humans. With transparent reporting, adequate power, and standardized endpoints, the field can define when PBM provides clinically meaningful WM benefit and how it should be integrated alongside established care.

**Table 1:** Preclinical PBM studies affecting microglia and white matter

Model/WM target	PBM regimen (λ, Power/Fluence, Mode)	Delivery Route/Schedule	Primary Cellular Effects (Microglia/Astrocyte)	WM & Functional Outcomes	Mechanistic Notes	Ref
Mouse SCI (model of white-matter tract injury)	808 nm (50 mW/cm², 150 J/cm², continuous wave)	Subcutaneous fiber irradiation over lesion; 1 h/day × 28 days	↓ injury-biased (M1-like) macrophage/microglia (iNOS, CD86, IL-1α/IL-6); ↑ miR-330-5p → STAT3↓	Neuroinflammation ↓; axon sparing ↑; motor recovery (BMS/LSS) ↑	Evidence: Supported. miR-330-5p targets STAT3, reducing M1-like polarization and supporting repair.	Bagnato et al., 2024
Rat T10 hemi-contusion SCI (model of white-matter tract injury and neuropathic pain)	670 nm LED (35 mW/cm²; ≈63.7 J/cm²; continuous)	Transcutaneous illumination to dorsal cord surface; 30 min/day × 7 days (start 2 h post-injury)	↓ iNOS⁺ IBA1⁺ microglia/macrophages; ↓ GFAP⁺ astrocyte reactivity (3–7 dpi); IL-1β unchanged	↓ neuronal apoptosis; no change in MBP or NF200; ↓ mechanical hypersensitivity incidence (1–5 dpi)	Evidence: Supported. iNOS downregulation in microglia and reduced astrogliosis are associated with pain attenuation independent of IL-1β.	Carneiro et al., 2019
Wistar rat T10 hemicontusion SCI (model of white-matter tract injury and neuropathic pain)	670 nm LED (35 mW/cm²; ≈63 J/cm²; continuous)	Transcutaneous illumination to dorsal cord surface; 30 min/day × 7 days (start 2 h post-injury)	↓ ED1⁺ microglia/macrophages; ↑ Arginase-1⁺ ED1⁺ repair-associated (M2-like) cells (~ 7× SCI); CD80⁺ injury-biased (M1-like) unchanged	↓ TUNEL⁺ cell death; ↓ pain hypersensitivity; ↑ dorsal column signal conduction and locomotor scores	Evidence: Supported (marker-based). Early increase in M2-like markers is associated with reduced cell death and improved white-matter and functional recovery.	Chan et al., 2021
Mouse T9 clip-compression SCI model (white-matter tract degeneration with macrophage/microglial activation)	808 nm (50 mW/cm², ≈ 150 J/cm², continuous wave)	Transcutaneous fiber-coupled irradiation to lesion area; 50 min/day × 28 days post-injury	↓ injury-biased (M1-like) macrophage polarization (iNOS, CD86, TNF-α, IL-6); ↑ miR-1192; ↓ lncRNA TUG1 and TLR3; NF-κB activation ↓	↑ neuronal survival and axon growth (DRG); ↑ motor function recovery (BMS, gait)	Evidence: Supported. TUG1–miR-1192–TLR3 axis downregulation is associated with reduced NF-κB signaling and reduced M1-like polarization, supporting SCI repair.	Chan et al., 2024
Mouse cuprizone-induced demyelination (corpus callosum)	808 nm GaAlAs laser; 50 mW; 1.78 W/cm² irradiance; 36 J/cm² fluence; continuous wave (single point 0.028 cm²)	Transcranial PBM; 3 consecutive days in week 3 + repeat in week 4 (total 6 sessions)	↓ IBA-1⁺ microglia number and perimeter (≈ 2300 vs. 5000 cells/mm² in CPZ); ↓ GFAP⁺ astrocytes number and perimeter (≈ 2500 vs. 3600 cells/mm²); ↑ OPCs (PDGFβR⁺/Ki67⁺ ≈ 2× CPZ)	↓ demyelination (LFB OD 91% vs. 79% in CPZ); ↑ MBP signal (51% vs. 20%); ↑ rotarod latency; LDH toxicity ↓ (trend)	Evidence: Supported for reduced microglia/astrocyte activation and increased OPC proliferation with improved myelin outcomes; inferred for CCO-mediated mitochondrial signaling.	Chao, 2019
Mouse T9 spinal cord compression injury (white-matter lesion with macrophage/microglial activation)	808 nm semiconductor laser; 50 mW/cm²; ~150 J/cm²; continuous wave	Implanted diffusing optical fiber at lesion surface; 50 min/day × 28 days	↓ iNOS⁺ CD86⁺ injury-biased (M1-like) macrophages; ↓ Notch1, HIF-1α, p-NF-κB; ↓ IL-1α, IL-6, COX-2; no effect on Arg1⁺ repair-associated (M2-like) cells	↑ motor recovery (BMS ↑, LSS ↑); ↓ neuronal apoptosis; ↑ DRG neurite length	Evidence: Supported. Notch1–HIF-1α–NF-κB axis downregulation is associated with reduced injury-biased polarization and cytokine output, supporting survival and recovery.	Chen et al., 2023
Mouse Experimental Autoimmune Encephalomyelitis (EAE, spinal white-matter demyelination)	670 nm LED (GaAlAs); 28 mW/cm²; 5 J/cm² per session; continuous wave (LED bandwidth 25–30 nm FWHM)	Whole-body transcutaneous irradiation (75 cm² array); 3 min/day × 10 days (suppression phase) and/or double-treatment regimen	↓ pro-inflammatory cytokines (IFN-γ, TNF-α); ↑ anti-inflammatory IL-4, IL-10 in lymph nodes and spinal cord; reduced Th1 response and microglial/macrophage activation	↓ clinical EAE score; sustained recovery in double-treatment group; ↑ remyelination and axonal integrity (implied by reduced oxidative stress and apoptosis)	Evidence: Supported for cytokine shift and reduced EAE severity; inferred for CCO/NO–mitochondrial photoacceptor pathway.	Cignarella et al., 2020
Rat T8 crush spinal-cord injury (white-matter tract degeneration with macrophage/microglial activation)	810 nm diode laser; 150 mW output; 0.3 cm² spot (≈ 0.5 W/cm², 900 J/cm² per session); continuous wave	Percutaneous vertical illumination to injury site; 50 min/day ×14 days	↓ injury-biased (M1-like) (iNOS⁺, CD86⁺) macrophage/microglia; ↑ repair-associated (M2-like) (Arg1⁺, CD206⁺) cells; ↑ IL-4 and IL-13 expression (ELISA) in spinal cord	↓ lesion and cavity area; ↑ neuronal survival (NeuN⁺); ↑ BBB locomotor scores at 7 & 14 dpi	Evidence: Supported (marker-based). Increased IL-4/IL-13 and M2-like markers with reduced M1-like markers; IL-4/IL-13–driven polarization is inferred.	Coleman & Höke, 2020
BALB/c mouse bilateral spinal cord compression SCI (model of white-matter degeneration and glial-scar formation)	810 nm diode laser (MW-GX-808); 500 mW/cm²; 1500 J/cm² per session; continuous wave (0.3 cm² spot)	Percutaneous vertical irradiation of lesion area; once daily for 1–21 days post-injury	↓ injury-biased (M1-like) (F4/80⁺ iNOS⁺) macrophages; ↓ GFAP⁺ astrocyte activation and CSPG (neurocan, NG2) expression; ↓ pSTAT3 in astrocytes	↑ motor recovery (BMS score ↑ at 7–21 dpi); ↓ glial scar formation around lesion	Evidence: Supported. Reduced M1-like macrophage markers and pSTAT3/CSPG with lower astrocyte activation, consistent with attenuated scar formation and repair.	de Freitas & Hamblin, 2016
Rat T10 clip-compression SCI (model of white-matter degeneration with neurotoxic microglia/astrocyte activation)	810 nm diode laser (MW-GX-808); 150 mW output; continuous wave; ≈ 0.5 cm² spot → ~300 mW/cm² surface irradiance (60 min/session × 14 days)	mplanted diffusing fiber fixed to lamina; irradiation initiated immediately post-injury, 60 min/day for 2 weeks	↓ iNOS⁺ Iba1⁺ microglia/macrophages; ↓ C3⁺ GFAP⁺ astrocytes; ↓ Lcn2, pJAK2, pSTAT3; ↓ IL-1α, C1q, TNF-α secretion; ↓ neurotoxic glial crosstalk	↑ NeuN⁺ motor neurons; ↓ TUNEL⁺ apoptosis; ↑ BBB and LSS scores at 7–28 dpi (behavioral recovery)	Evidence: Supported. Reduced Lcn2/JAK2–STAT3 and neurotoxic microglia–astrocyte signatures are associated with reduced secondary damage and improved recovery.	Deczkowska et al., 2018
Rat T10 bilateral clamp SCI (model of white-matter degeneration and neuropathic pain)	810 nm diode laser; 150 mW output; continuous mode; ≈ 0.5 cm² spot → ~300 mW/cm²; ~540 J per session	Implanted fiber fixed to T9–T10 lamina; 60 min/day × 14 days starting immediately post-injury	↓ CXCL10 and CXCR3 expression in Iba1⁺ microglia and GFAP⁺ astrocytes; ↓ p-NF-κB (p-P65); ↓ TNF-α, IL-1β, IL-6, IL-18; ↑ IL-10 in spinal cord	↓ mechanical allodynia, cold allodynia, heat hyperalgesia; ↑ BBB locomotor score (7–28 dpi) indicating functional WM recovery	Evidence: Supported. Reduced NF-κB activation and CXCL10/CXCR3 signaling in glia is associated with attenuated neuroinflammation and neuropathic pain after SCI.	Ding et al., 2024
Mouse T9 clamp spinal-cord injury (white-matter degeneration with macrophage/microglial activation)	808 nm laser (MW-GX-808); 50 mW/cm²; 150 J/cm²; continuous wave (5 nm bandwidth)	Implanted diffusing fiber targeting lesion; 50 min/day × 28 days post-injury	↓ injury-biased (M1-like) macrophage markers (F4/80⁺ iNOS⁺, CD86⁺); ↓ TLR2, NLRP3, Caspase-1, IL-1β; ↑ LC3B, Beclin-1; ↓ p62; enhanced autophagy in macrophages	↑ BMS and LSS scores (7–28 dpi); improved hind-limb gait and stride length; ↓ inflammatory cytokines (IL-1β, IL-6, TNF-α, COX-2)	Evidence: Supported. Reduced TLR2-dependent NLRP3/Caspase-1/IL-1β signaling and restored autophagy markers are associated with improved white-matter repair and functional recovery.	Duarte et al., 2018

NR, not reported. “Evidence” tags in the Mechanistic Notes column indicate the strength of mechanistic support within each cited study: Supported denotes evidence linked to functional assays and/or coherent pathway–phenotype–outcome readouts within the same model, whereas Inferred denotes mechanisms proposed mainly from marker/pathway changes or indirect evidence without direct causal testing (e.g., photoacceptor attribution)

**Table 2:** Clinical PBM studies with white‑matter–relevant endpoints

Study Design/*N*	Target Condition	PBM Parameters (λ, Power/Fluence, Mode)	Delivery Site/Duration	WM-Related Imaging or Cognitive Endpoints	Key Outcomes/Remarks	Ref
Randomized, double-blind, sham-controlled clinical trial; *n* = 68 (33 active/35 sham; 43 completed MRI follow-up)	Moderate traumatic brain injury (TBI) within 72 h	λ = 810 nm LED array (reported 600–1100 nm range) Mode: continuous wave Incident fluence: ≈ 43 J/cm² per 20‑min session Estimated cortical dose: ≈ 1.3 J/cm² (authors’ model) Irradiance/spot size/coverage: NR	Helmet with 18 LED clusters 3 × 20 min sessions within 72 h post‑injury (≈ 12 h interval)	WM imaging: diffusion MRI (FA, MD, RD, AD) across 18 major WM tracts (acute; 2–3 wk; 3 mo) Microglial/inflammation biomarker: None/NR Clinical: Rivermead Post‑Concussion Questionnaire (RPQ)	Safe and feasible. Significant time × treatment interactions in diffusion metrics at 3 mo (RD *P* < 0.001; MD *P* = 0.03; FA *P* < 0.001). RPQ symptom scores showed a non‑significant trend.	Bagnato et al., 2024
Prospective clinical study; *n* = 10 (chronic severe TBI; 4 mo–4 year post‑injury)	Chronic traumatic brain injury with diffuse axonal injury and/or intracranial hemorrhage	λ = 630 nm LED Mode: continuous wave Irradiance: 25.7 mW/cm² Fluence: ≈ 3.7 J/cm² per 30‑min session (as reported) Spot size/intracranial dose: NR	Whole‑scalp helmet‑type device (~ 400 cm²) 30 min/session; 3×/week × 6 weeks (18 sessions)	WM imaging: None/NR Microglial/inflammation biomarker: None/NR Vascular: transcranial Doppler (CBF, PSV, MFV, PI) Cognitive: neuropsychological battery (Rey figure, TMT A/B, Stroop, RAVLT, F‑A‑S)	↑ left MCA peak systolic velocity (*p* = 0.007). Improvements reported across multiple cognitive domains and depressive symptoms. Safe and well‑tolerated (non‑randomized; small n).	Carneiro et al., 2019
Randomized, sham-controlled study; *n* = 18 (older adults with MCI)	Mild cognitive impairment (MCI)	λ = 810 nm LED Mode: continuous wave Irradiance: 20 mW/cm² Fluence: 7 J/cm² Single session: 350 s	Forehead stimulation (F7, AF7, Fp1, Fpz, Fp2, AF8, F8, Fz, Cz) Single session (350 s)	WM imaging: None/NR Microglial/inflammation biomarker: None/NR Functional: fNIRS (oxy‑Hb/deoxy‑Hb) during visual memory span task Cognitive: visual memory span performance	Visual memory span improved by 29.3% (*p* = 0.05). Reduced frontal hemodynamic response across channels (*p* < 0.05–0.01) was reported.	Chan et al., 2021
Randomized, double-blind, placebo-controlled trial; *n* = 60 (40 active/20 sham; 57 completed)	Mild-to-moderate dementia (mixed etiologies)	λ = 1060–1080 nm LEDs Mode: continuous wave Irradiance: 23.1 mW/cm² over ~ 650 cm² Session duration: 6 min; twice daily × 8 weeks Approx fluence: ≈ 8.3 J/cm² per session (calculated from irradiance × time) Intracranial dose: NR	Helmet (12 cranial modules + 2 ocular modules) 6 min/session; twice daily × 8 weeks; self‑administered at home	WM imaging: None/NR Microglial/inflammation biomarker: None/NR Cognitive: neuropsychological battery (MMSE; Logical Memory I/II; TMT A/B; Boston Naming; AVLT; Digit Span; Word Fluency)	Active group showed improvements across multiple cognitive measures (e.g., MMSE + 4.8; *p* < 0.001). No adverse events reported. Interpretation is limited by mixed dementia subtypes and short follow‑up.	Chan et al., 2024
Randomized parallel‑group controlled pilot; *n* = 8 (4 PBM/4 usual care)	Mild-to-moderate dementia	λ = 810 nm LEDs (transcranial + intranasal) Mode: pulsed 40 Hz; 50% duty cycle Device outputs reported (posterior 100 mW × 3; anterior 75 mW × 2; intranasal 25 mW × 1) Per‑session fluence/irradiance at scalp: NR Session duration: 20 min (reported total energy ≈ 240 J)	Transcranial + intranasal 3 sessions/week × 12 weeks (home use)	WM imaging: None/NR Microglial/inflammation biomarker: None/NR Perfusion: ASL‑MRI (cortical ROIs) Functional: resting‑state fMRI (DMN connectivity) Cognitive/behavior: ADAS‑Cog; NPI	↑ cerebral perfusion in reported ROIs (*p* < 0.03) and ↑ DMN connectivity (*p* < 0.01). ADAS‑Cog improved (− 5.2 points) and NPI decreased (− 22.8 points). Pilot study; underpowered; no adverse events.	Chao, 2019
Prospective, double-blind, sham-controlled RCT (secondary imaging analysis); *n* = 38 (17 active/21 sham) + 23 controls	Moderate TBI within 72 h	λ = 810 nm NIR helmet (bandwidth 30 nm) Mode: continuous wave Session duration: 20 min × 3 sessions within 72 h Irradiance/fluence/intracranial dose: NR (not provided in this table)	Whole‑head LED helmet 3 sessions within 72 h; separated by ≥ 12 h	WM imaging: None/NR Microglial/inflammation biomarker: None/NR Functional: resting‑state fMRI connectivity (82‑region RSFC matrix; acute ≤ 1 wk, 2–3 wk, 3 mo) Clinical: RPQ	↑ RSFC in 7 region pairs during acute→subacute phase in active vs. sham (FDR *p* = 0.01–0.047). No RPQ difference reported.	Chen et al., 2023

NR, not reported. WM-specific imaging endpoints were uncommon across available clinical PBM studies; only one randomized trial in moderate TBI reported diffusion-MRI white-matter metrics. Microglial PET or other microglia-specific biomarkers were not included in these studies and remain a key gap for translation

**Table 3:** Mechanistic links between white-matter pathology and PBM

Pathophysiological node	Mechanism	Representative PBM evidence	WM-relevant effect	Ref
Neuroinflammation (microglia/astrocyte toxicity)	Evidence: Supported. PBM associated with reduced Lcn2–JAK2/STAT3 signaling and lower neurotoxic glial signatures; apoptosis reduced in this model.	Rat SCI; 810 nm; 150 mW; 60 min/day × 14 d; epidural/implant fiber.	Direct WM relevance: tissue sparing ↑; apoptosis ↓; locomotor recovery ↑ (spinal WM injury model).	Bagnato et al., 2024
Demyelination/remyelination (toxin-induced)	Evidence: Supported. Reduced activated microglia/astrocytes and increased OPC-lineage markers reported alongside improved myelin histology.	Mouse cuprizone; 808 nm; CW; 36 J/cm²/session; 6 sessions; transcranial.	Direct WM endpoint: demyelination ↓ (LFB); MBP/OL markers ↑; motor performance ↑.	Carneiro et al., 2019
Traumatic diffuse injury (TBI/CCI)	Evidence: Supported (microglial activation). Early reduction of Iba1⁺ microglial activation reported; mechanistic links to WM repair not directly tested.	Mouse CCI; 800 nm; 60 J/cm²; transcranial; schedule NR.	WM relevance: Indirect—primarily cognitive outcome (MWM) with microglial marker changes; WM-specific endpoints NR.	Chan et al., 2021
Amyloid pathology with microglial engagement	Evidence: Supported (Aβ engagement). Increased microglia–Aβ contact and reduced plaque burden reported; WM effects remain indirect unless WM endpoints are measured.	AD mouse; 1070 nm; 10 Hz pulsed; transcranial; irradiance/fluence/schedule NR in summary.	WM relevance: Indirect—network-level benefit plausible, but WM-specific endpoints NR in this entry.	Chan et al., 2024
Perfusion/endothelial NO signaling (vascular support)	Evidence: Supported (vascular signaling). PBM associated with eNOS phosphorylation and increased CBF in stroke preconditioning; direct WM endpoints not reported here.	Mouse stroke preconditioning; 1064 nm; 50 mW/cm²; other dosimetry NR.	WM relevance: Indirect—improved perfusion may support WM vulnerability, but WM-specific imaging/histology NR.	Chao, 2019
Neuroinflammation and microglial phenotype bias (SCI)	Evidence: Supported (phenotype + outcomes). Reported shift toward repair-associated (M2-like) markers with reduced NF-κB/oxidative stress; interpretation remains context- and marker-dependent.	Mouse SCI; 810 nm; 150 mW/cm²; 20 min/day × 14 d; transcutaneous dorsal.	Direct WM endpoints: MBP ↑; axon sparing ↑; locomotor recovery ↑.	Chen et al., 2023
Inflammation-associated axonal degeneration in MS model (EAE)	Evidence: Supported. PBM reduced glial reactivity and peripheral immune infiltration and prevented axonal loss in spinal WM in this model.	Mouse EAE; 660 + 850 nm; 10 Hz, 50% duty; 6 min/day × 17 d; 8.86–18.5 mW/cm²; dorsal + ventral.	Direct WM endpoint: axonal density ↑/axonal loss prevented; Iba1⁺/GFAP⁺ reactivity ↓; sensorimotor function ↑.	Cignarella et al., 2020
Myelin/OL differentiation signaling (AKT1/mTOR) in PND	Evidence: Supported (pathway + myelin markers). PBM reported to activate AKT1/mTOR and increase OLIG2/MBP; remyelination is inferred from marker changes unless structural WM endpoints are provided.	Aged PND mouse; 810 nm; 120 mW/cm²; 18 J/cm²; 2.5 min/day × 3 d; transcranial.	WM relevance: Indirect-to-moderate—OLIG2/MBP ↑; cognitive recovery ↑; structural WM imaging/histology NR in this entry.	Coleman & Höke, 2020
Microglial metabolic reprogramming & oxidative stress (Aβ challenge, in vitro)	Evidence: Supported (cellular metabolism/phagocytosis). PBM restored Δψm/ATP, reduced ROS/cytokines, and increased Aβ phagocytosis in vitro; in vivo WM translation remains inferential.	Primary mouse microglia + Aβ1–42; 808 nm; 30 mW/cm² × 5 min (≈ 10 J/cm²); single CW in vitro.	WM relevance: Indirect—mechanistic support for microglial energy homeostasis and phagocytosis; WM endpoints not applicable (in vitro).	de Freitas & Hamblin, 2016
Microglial phenotype switch & inflammasome (PND)	Evidence: Supported (microglial markers + inflammation). PBM upregulated IRF7, reduced NLRP3 activation and inflammatory mediators; myelin-specific endpoints NR.	Aged PND mouse; 810 nm LED; 80 mW/cm²; 200–400 s/day × 5 d; transcranial.	WM relevance: Indirect—neuroinflammation ↓; BDNF/synaptic markers ↑; cognitive performance ↑; WM endpoints NR.	Deczkowska et al., 2018

NR, not reported. Evidence tags indicate whether the cited study provides supportive functional/pathway–phenotype–outcome linkage within the same model (Supported) or whether the mechanistic link to WM repair remains indirect/marker-based (Indirect/Inferential). WM relevance is labeled as direct when WM-specific histology/imaging or tract/axonal endpoints are reported, and indirect when conclusions rely on behavioral/vascular/network outcomes without WM-specific measures

## Data Availability

No datasets were generated or analysed during the current study.
